# On the localization of high-frequency, sinusoidally amplitude-modulated tones in free field

**DOI:** 10.1121/1.4976047

**Published:** 2017-02-15

**Authors:** Eric J. Macaulay, Brad Rakerd, Thomas J. Andrews, William M. Hartmann

**Affiliations:** Department of Physics and Astronomy, Michigan State University, 567 Wilson Road, East Lansing, Michigan 48824, USA; Department of Communicative Sciences and Disorders, Michigan State University, 1026 Red Cedar Road, East Lansing, Michigan 48824, USA; Department of Physics and Astronomy, Purdue University, 525 Northwestern Avenue, West Lafayette, Indiana 47907, USA; Department of Physics and Astronomy, Michigan State University, 567 Wilson Road, East Lansing, Michigan 48824, USA

## Abstract

Previous headphone experiments have shown that listeners can lateralize high-frequency sine-wave amplitude-modulated (SAM) tones based on interaural time differences in the envelope. However, when SAM tones are presented to listeners in free field or in a room, diffraction by the head or reflections from room surfaces alter the modulation percentages and change the shapes of the envelopes, potentially degrading the envelope cue. Amplitude modulation is transformed into mixed modulation. This article presents a mathematical transformation between the six spectral parameters for a modulated tone and six mixed-modulation parameters for each ear. The transformation was used to characterize the stimuli in the ear canals of listeners in free-field localization experiments. The mixed modulation parameters were compared with the perceived changes in localization attributable to the modulation for five different listeners, who benefited from the modulation to different extents. It is concluded that individual differences in the response to added modulation were not systematically related to the *physical* modulation parameters themselves. Instead, they were likely caused by individual differences in *processing* of envelope interaural time differences.

## I. INTRODUCTION

In the late 1950s, psychoacousticians discovered that listeners could lateralize a high-frequency tone with no interaural level difference (ILD) on the basis of a modulated envelope. A review of the literature indicates intense parallel efforts by the group at Bell Labs (David *et al.*, 1958, 1959) and the group at Imperial College (Leakey *et al.*, 1958). It was found that tones could be lateralized on the basis of interaural time differences (ITD) even if their frequencies were so high that no fine-structure ITD was perceptually available. Instead, listeners were able to use envelope interaural time differences (EITD). Starting in the 1970s, the effect was further developed in another round of parallel transatlantic efforts (Henning, 1974, 1980, 1983; McFadden and Pasanen, 1976; Bernstein and Trahiotis, 1985a, 1985b). All of these experimental studies used headphones for stimulus presentation. One of the interesting features afforded by headphones was the opportunity to use different carrier frequencies in the two ears, but a common amplitude modulation. Such experiments then focused on the idea that a common modulation could lead to binaural fusion of signals made with somewhat different carrier frequencies.

Towards the 21st century, a third round of transatlantic efforts again used headphone experiments to study the effects of different types of modulation both on the lateralization and on the binaural advantages of modulated stimuli, focussing particularly on “transposed stimuli,” where high-frequency sine tones were given envelopes or other structure to mimic low-frequency waveforms as transduced by the peripheral auditory system (van de Par and Kohlrausch, 1997; Bernstein and Trahiotis, 2002, 2003; Majdak and Laback, 2009). More recent psychoacoustical experiments have performed a microscopic analysis of on-going envelope features, particularly onsets, and related these to physiological observations (Klein-Hennig *et al.*, 2011; Laback *et al.*, 2011; Francart *et al.*, 2012; Dietz *et al.*, 2015; Dietz *et al.*, 2016). Interest in the EITD as a localization cue was particularly stimulated by the realization that it is the only temporal cue to localization that is available with contemporary cochlear implant coding (van Hoesel and Tyler, 2003; van Hoesel *et al.*, 2009).

Macaulay *et al.* (2010) employed an alternative to headphone listening. Their experiments explored the free-field localization of high-frequency tones in an anechoic room. The tone frequency was high enough that listeners could not use fine-structure ITDs to localize. Instead, they could only use ILDs. Experiments with unmodulated tones found a major disruption of sound localization caused by the acoustical bright spot. The bright spot caused the ILD to be a non-monotonic function of azimuth (Kuhn, 1977), which led to large localization errors for pure tones. However, further experiments showed that adding low-frequency (100-Hz) amplitude modulation to the tones, with consequent low-frequency interaural time differences in the envelope, allowed some, but not all, listeners to circumvent the confusion caused by the bright spot and to localize correctly over an entire quadrant of azimuths. These experiments showed that the information in modulation was not limited to lateralization of tones presented by headphones but could also be beneficial for localization in free field, at least for some listeners.

An amplitude-modulated (AM) tone presented in free field is different from an AM tone presented by headphones. With headphone presentation, there is good reason to believe that the signals in the left and right ear canals retain the character of the original stimuli as computed or otherwise electronically generated. (Further evidence is reported below in Sec. IV E.)

**FIG. 1.**
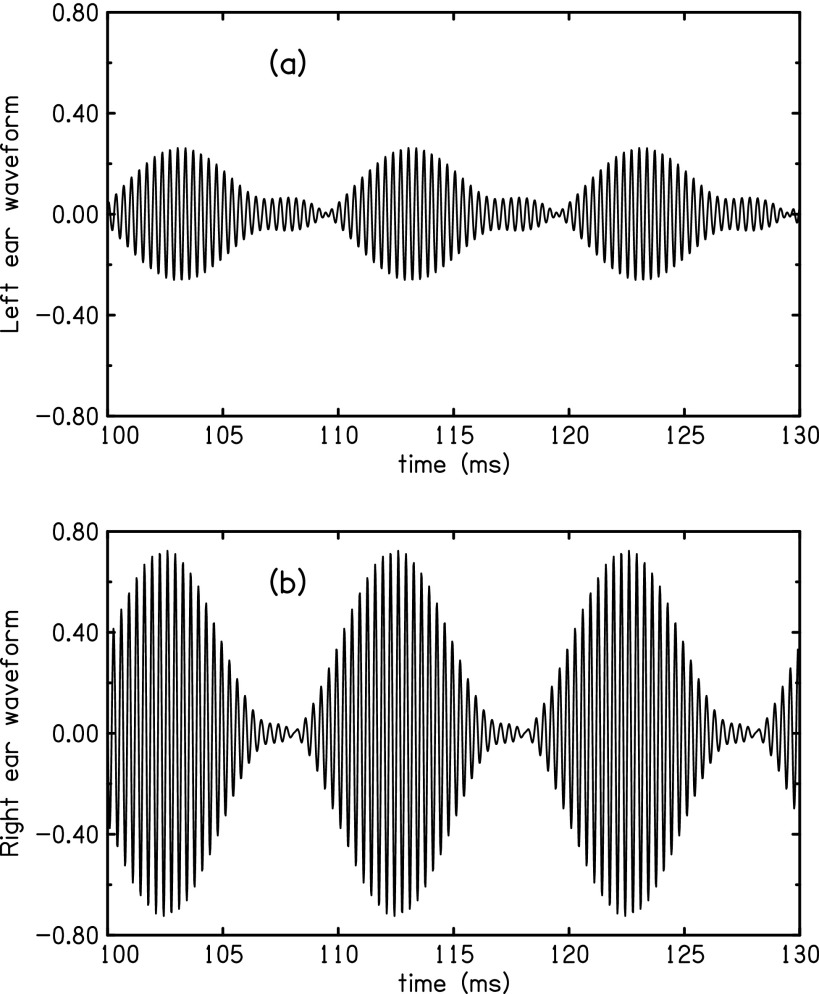
Signals measured in the (a) far (left) and (b) near (right) ear canals of a listener given a 3000-Hz, AM tone with 100% modulation at 100 Hz, presented by a loudspeaker at 90° azimuth—the extreme right side of the listener. Levels and shapes are different at the two ears. Vertical scales are the same in parts (a) and (b) but arbitrary. Modulation fractions (*m*) are, respectively, 1.38 and 1.09. QFM indices (*β*) are, respectively, 0.56 and 0.20.

By contrast, when an AM tone is presented through loudspeakers, diffraction by the listener's head causes changes in the modulation. For example, if the original AM signal is 100% modulated, the modulation in an ear canal may be less than 100% or it may be more (over modulation) due to the frequency dependence of the transfer function from the loudspeaker to the ear. Irregularities in the response of the loudspeaker itself may also contribute. Inevitably, the envelope peaks and valleys will have different heights and depths in the two ears, and AM will be converted into a mixed modulation including quasi-frequency modulation (QFM). Because the envelopes in the two ears may have different shapes, it may be difficult for the binaural system to identify corresponding features in the left and right envelopes. That would complicate the process of determining an EITD. For example, Fig. 1 shows the waveforms measured in a listener's ear canals for a 3000-Hz tone having 100%, 100-Hz amplitude modulation as delivered to a loudspeaker at 90° of azimuth. Clearly, the envelopes are differently shaped. The problem is to know what aspects of these different shapes should be compared in time in order to use the EITD to localize.

**FIG. 2.**
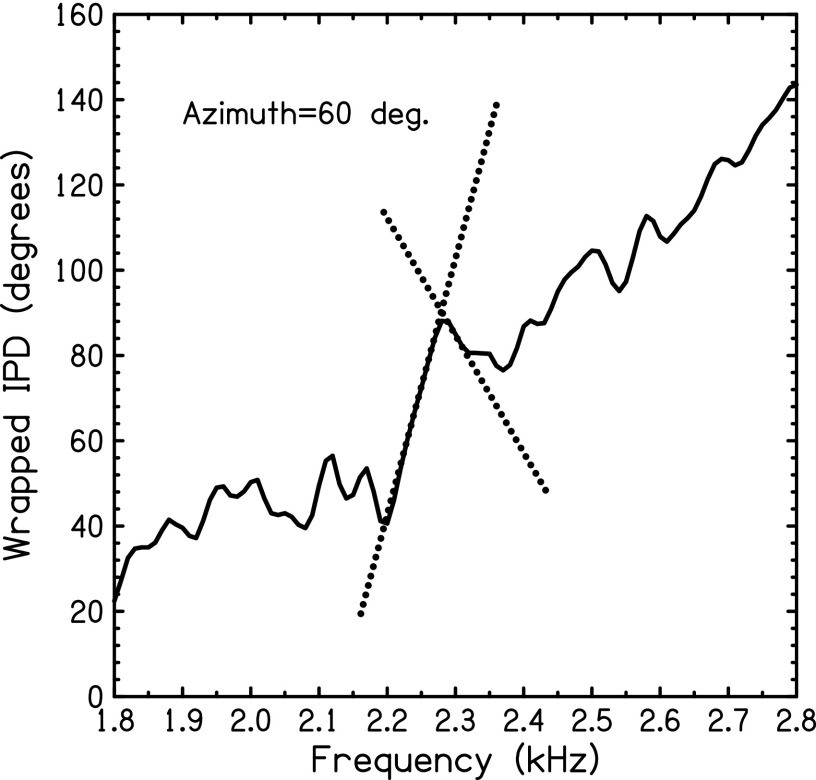
IPDs measured between the ear canals for a sine tone source at 60° azimuth in free field. The slope of this function is the group delay, and two particular slopes are shown by dotted lines illustrating an abnormally large positive group delay and a negative group delay. The frequency ranges for these two effects are smaller than the 200-Hz range for the experiments in this article.

Finally, there is a fundamental difference between the ITD and the EITD as they appear in free field. The ITD depends on the phase delay of the signal as it is diffracted around the head. The phase delay has an unambiguous sign. The EITD is related to the group delay of the signal, which is determined by the slope of the interaural phase difference (IPD) as a function of frequency. Because diffraction can lead to a slope that is opposite in sign to the phase shift itself, the group delay sometimes results in an EITD cue that points to a side opposite to the ITD and opposite to the source. A sample IPD measured in ear canals for a source at 60° of azimuth, is shown in Fig. 2. Two slopes are noted: The positive slope is 1667 *μ*s—a group ITD far larger than the physiological limit for human heads. The negative slope is −764 *μ*s, and it points to a source at about 90° azimuth on the opposite side of the head. Both of these group delays would be highly misleading cues for localization. A detailed treatment of group delay, as it applies to modulated signals appears in Appendix A.

The present report continues the study of the free-field localization of AM tones begun by Macaulay *et al.* (2010). It deals with the complexities of the EITD for a modulated signal that has been diffracted around the head, and with the effects of these complexities on the human ability to use EITD to localize sounds. Specifically, this report tries to determine whether individual differences in accessing the information in the EITD can be attributed to individual differences in sound wave diffraction. There are two parts to this report: The first part presents mathematical formulae by which the amplitude and phase spectra of a modulated signal, as measured in the ear canal, can be converted into mixed modulation parameters. This mathematical transformation is useful in any context where modulated signals are linearly distorted, and to the best of our knowledge, it has not previously been derived or discussed. The second part of this report uses the mixed modulation parameters measured in the ear canals of five listeners to compare with the localization decisions made by those listeners in free-field experiments. In this way, it serves as an initial microscopic analysis of sound localization by interaural envelope timing as it occurs in real-world conditions. It is particularly relevant to localization by cochlear implantees for nearby sources where the real world might be approximated by free field.

## II. SPECTRUM TRANSFORMATION

**FIG. 3.**
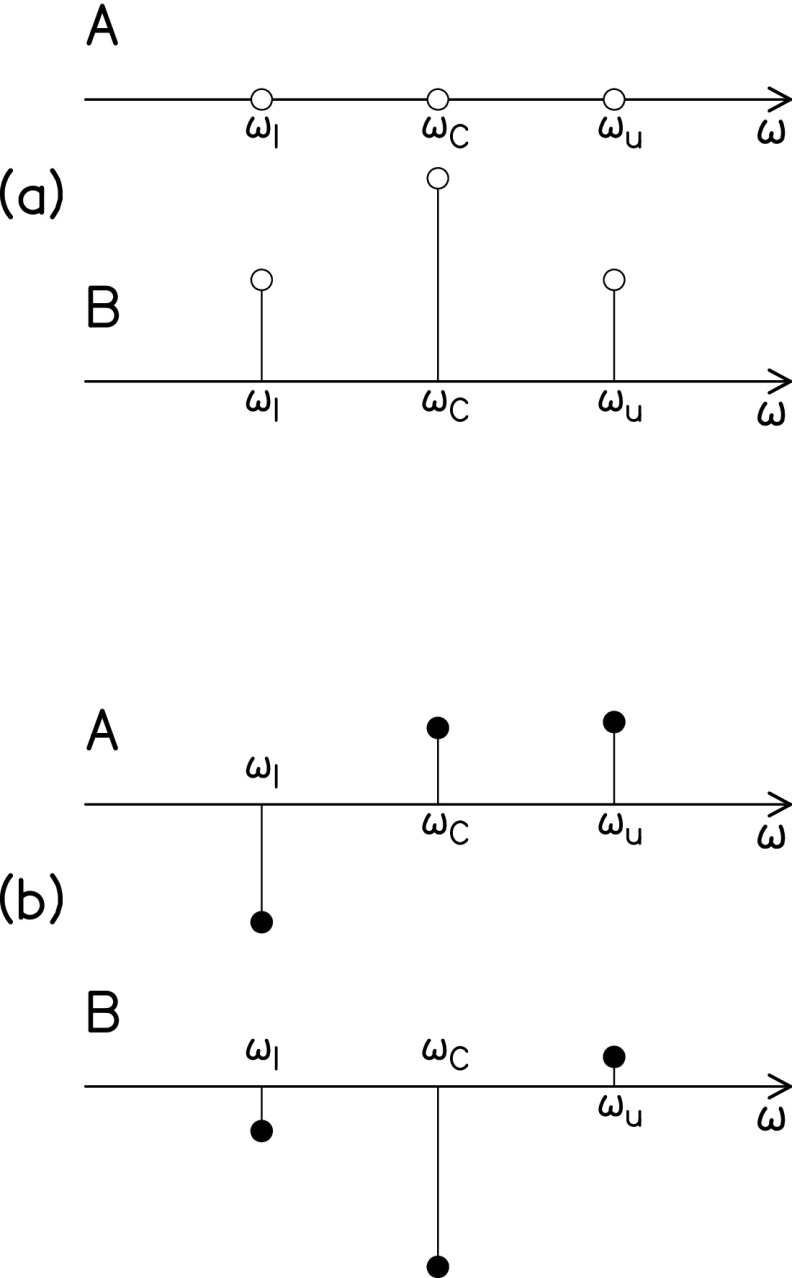
(a) Carrier and sidebands in the spectrum of a 100% amplitude modulated tone. Symbols A and B refer to cosine and sine spectral components, respectively. (b) The six spectral components that were obtained by Fourier transforming the right ear canal signal from Fig. 1.

An AM signal, as sent to a loudspeaker in our experiments, is a purely AM signal,^1^

$$x_{o}(t)=C[1+m\;\text{cos}(\omega_{m}t+\phi_{a})]\;\text{sin}(\omega_{c}t+\phi_{c}),$$ (1)

where *m* represents the modulation fraction and subscripts *c* and *m* stand for “carrier” and “modulation.” The signal has two side bands, a lower sideband, having angular frequency $\omega_{\ell }$, given by subtracting the modulation frequency from the carrier frequency, $\omega_{\ell }$ = *ω_c_* – *ω_m_*, and an upper sideband at *ω_u_* = *ω_c_* + *ω_m_*, as shown in Fig. 3(a).

### A. Mixed modulation

Because of head diffraction (or room reflections, if present), the signal in an ear canal is no longer entirely AM but includes a frequency modulation (FM) component. However, because the acoustical transformations are linear, there are still only two sidebands. Therefore, the FM component is QFM. Together, the AM and QFM is a form of mixed modulation (Hartmann and Hnath, 1982; Edwards and Viemeister, 1994; Hartmann, 1998). Further, it is a mathematical fact that any combination of carrier and sideband amplitudes and phases can be uniquely represented by a mixed modulation signal of the form,

$$x(t)=C[1+m\;\text{cos}(\omega_{m}t+\phi_{a})]\;\text{sin}(\omega_{c}t+\phi_{c})+C\beta \;\text{sin}(\omega_{m}t+\phi_{f})\;\text{cos}(\omega_{c}t+\phi_{c}).$$ (2)

Here, the first two terms represent the carrier and AM component, and the term in *β* represents the QFM. Parameter *β* is the modulation index, and $\phi_{f}$ is the phase of the QFM. It is defined such that, if $\phi_{f}=\phi_{a}$ the maximum (minimum) in frequency occurs when the maximum (minimum) in amplitude occurs. The modulation index is equal to Δ*ω*/*ω_m_*, where Δ*ω* is a frequency excursion, equal to half the peak-to-peak excursion.

Expanding the products of sines and cosines in Eq. (2) leads to the two sidebands in the spectrum. The sidebands have the same frequencies as for pure AM, but the relative amplitudes and phases are changed by the acoustical situation.^2^

Formally, *x*(*t*) can be written in terms of Fourier components,

$$x(t)=A_{c}\;\text{cos}\;\omega_{c}t+B_{c}\;\text{sin}\;\omega_{c}t+A_{\ell }\;\text{cos}\;\omega_{\ell }t+B_{\ell }\;\text{sin}\;\omega_{\ell }t+A_{u}\;\text{cos}\;\omega_{u}t+B_{u}\;\text{sin}\;\omega_{u}t,$$ (3)

where a pair of coefficients (*A* and *B*) is an alternative to an amplitude and phase form. The virtue of the *A* and *B* coefficients is that they can be easily determined from an ear canal signal, *x*(*t*). For instance, for the carrier,

$$A_{c}=\frac{2}{T}\int_{0}^{T}dt\;x(t)\text{cos}\;\omega_{c}t,$$ (4)

and

$$B_{c}=\frac{2}{T}\int_{0}^{T}dt\;x(t)\text{sin}\;\omega_{c}t,$$ (5)

where *T* is the duration of signal *x*(*t*). Coefficients for lower and upper sidebands, $A_{\ell }$, $B_{\ell }$, *A_u_*, and *B_u_* can be determined from the same equations with subscripts ℓ and *u* replacing subscript *c*. Analyzing the modulated signals through these integrals is equivalent to an ideal form of matched filtering—eliminating noise and interference—because the relevant frequencies are known exactly. Determination of the modulation parameters through these spectral coefficients is better than fitting the modulation in the waveform because there are more cycles in the carrier and sidebands than in the modulation itself.

Therefore, our procedure began with six Fourier coefficients, as shown in Fig. 3(b). An inspection of Eq. (2) shows that there are also six mixed modulation parameters, [$C,\phi_{c},m,\phi_{a},\beta$ and $\phi_{f}]$. Expanding the functions in Eq. (2) leads to the following relationships:

$$A_{c}=C\;\text{sin}\;\phi_{c},$$ (6a)

$$B_{c}=C\;\text{cos}\;\phi_{c},$$ (6b)

$$A_{\ell }/C=\frac{1}{2}\left[m\;\text{sin}(\phi_{c}-\phi_{a})-\beta \;\text{sin}(\phi_{c}-\phi_{f})\right],$$ (6c)

$$B_{\ell }/C=\frac{1}{2}\left[m\;\text{cos}(\phi_{c}-\phi_{a})-\beta \;\text{cos}(\phi_{c}-\phi_{f})\right],$$ (6d)

$$A_{u}/C=\frac{1}{2}\left[m\;\text{sin}(\phi_{c}+\phi_{a})+\beta \;\text{sin}(\phi_{c}+\phi_{f})\right],$$ (6e)

$$B_{u}/C=\frac{1}{2}\left[m\;\text{cos}(\phi_{c}+\phi_{a})+\beta \;\text{cos}(\phi_{c}+\phi_{f})\right].$$ (6f)

It is possible to invert these equations to find the six mixed modulation parameters in terms of the six measured Fourier coefficients by the decomposition described in Sec. II B.

### B. Decomposition into AM and QFM

Because the component at the carrier frequency does not involve any modulation, it is easy to solve for parameters *C* and ϕ_*c*_,

$$C=\sqrt{A_{c}^{2}+B_{c}^{2}},$$ (7)

and

$$\text{tan}(\phi_{c})=A_{c}/B_{c}.$$ (8)

Carrier phase $\phi_{c}$ can be obtained by inverting Eq. (8). This phase, as well as phases $\phi_{a}$ and $\phi_{f}$, need to be determined from the **Arg** function because the inverse tangent function is restricted to the principal values between –*π*/2 to *π*/2, but the phase needs to be computed over the full range, –*π* to *π*.

Solving for the other parameters requires much more algebra. The answers for the AM and QFM phases are, respectively,

$$\text{tan}(\phi_{a})=\frac{(A_{u}-A_{\ell })\text{cos}\;\phi_{c}-(B_{u}-B_{\ell })\text{sin}\;\phi_{c}}{(B_{u}+B_{\ell })\text{cos}\;\phi_{c}+(A_{u}+A_{\ell })\text{sin}\;\phi_{c}}$$ (9)

and

$$\text{tan}(\phi_{f})=\frac{(A_{u}+A_{\ell })\text{cos}\;\phi_{c}-(B_{u}+B_{\ell })\text{sin}\;\phi_{c}}{(B_{u}-B_{\ell })\text{cos}\;\phi_{c}+(A_{u}-A_{\ell })\text{sin}\;\phi_{c}}.$$ (10)

Solving these equations requires that the carrier phase $\phi_{c}$ be first calculated from Eq. (8) above. Following the steps of the solution makes it evident that if AM fraction *m* is zero, the right hand side of Eq. (9) is 0/0 and the solution for $\phi_{a}$ is indefinite, reflecting the fact that there is no point to an AM phase if there is no AM. Further, the denominator of Eq. (9) is zero only if *m* = 0. Similarly, if modulation index *β* is zero, the right hand side of Eq. (10) is 0/0 and the solution for the QFM phase, $\phi_{f}$, is indefinite. Further, the denominator of Eq. (10) is zero only if *β* = 0.

Having found the modulation phases ($\phi_{a}$ and $\phi_{f}$), it is possible to find the modulation fraction *m*.

$$m=\frac{(B_{u}+B_{\ell })\text{cos}\;\phi_{c}+(A_{u}+A_{\ell })\text{sin}\;\phi_{c}}{C\;\text{cos}\;\phi_{a}}$$ (11)

or

$$m=\frac{(A_{u}-A_{\ell })\text{cos}\;\phi_{c}-(B_{u}-B_{\ell })\text{sin}\;\phi_{c}}{C\;\text{sin}\;\phi_{a}}.$$ (12)

In the general case, either Eq. (11) or Eq. (12) may be used. If a denominator in one of those equations happens to be zero, the other equation should be used.

It is possible to find the QFM index, *β*,

$$\beta =\frac{(B_{u}-B_{\ell })\text{cos}\;\phi_{c}+(A_{u}-A_{\ell })\text{sin}\;\phi_{c}}{C\;\text{cos}\;\phi_{f}}$$ (13)

or

$$\beta =\frac{(A_{u}+A_{\ell })\text{cos}\;\phi_{c}-(B_{u}+B_{\ell })\text{sin}\;\phi_{c}}{C\;\text{sin}\;\phi_{f}}.$$ (14)

In the general case, either Eq. (13) or Eq. (14) may be used. If a denominator in one of those equations is zero, the other equation should be used.

Although the QFM formula is often presented as an approximation for FM in communications text books (narrow-band FM), the decomposition of the diffraction-distorted AM signal into the combination of AM and QFM, as defined above, is exact. It is a complete solution for the general case of a carrier and two sidebands, and that exhausts the possibilities for a linearly distorted AM signal.

### C. Modulation of the envelope

The decomposition into AM and QFM in Sec. II B represents the modulation of the amplitude by fraction *m*. However, it is expected that listeners will be sensitive to the modulation of the *envelope* of *x*(*t*), which is not exactly the same thing. The reason is that QFM itself has an AM component. The difference between *m* and the modulation in the envelope can be examined by writing envelope *E* in terms of *A* and *B* parameters. Envelope *E* is found by beginning with Eq. (3) for *x*(*t*) and computing the Hilbert transform $\hat{x}(t)$. Then $E^{2}(t)=x^{2}(t)+{\hat{x}}^{2}(t)$, or

$$E^{2}(t)={\left[A_{c}+(A_{u}+A_{\ell })\;\text{cos}\;\omega_{m}t+(B_{u}-B_{\ell })\;\text{sin}\;\omega_{m}t\right]}^{2}+{\left[B_{c}+(B_{u}+B_{\ell })\;\text{cos}\;\omega_{m}t+(A_{\ell }-A_{u})\;\text{sin}\;\omega_{m}t\right]}^{2}.$$ (15)

The envelope of interest is the square root of Eq. (15). An analysis of Eq. (15) shows that the contribution to the modulation of the envelope caused by the QFM is second order in *β*. Consequently, the frequency of this contribution is 2*ω_m_* and not *ω_m_* like the AM part of the decomposition. A contribution at a rate of 2*ω_m_* can clearly be seen in the top panel of Fig. 1. Because there is no first order term in *β*, the modulation fraction, *m*, calculated from the decomposition is a reasonable approximation to the envelope modulation so long as *β* does not become too large.

**FIG. 4.**
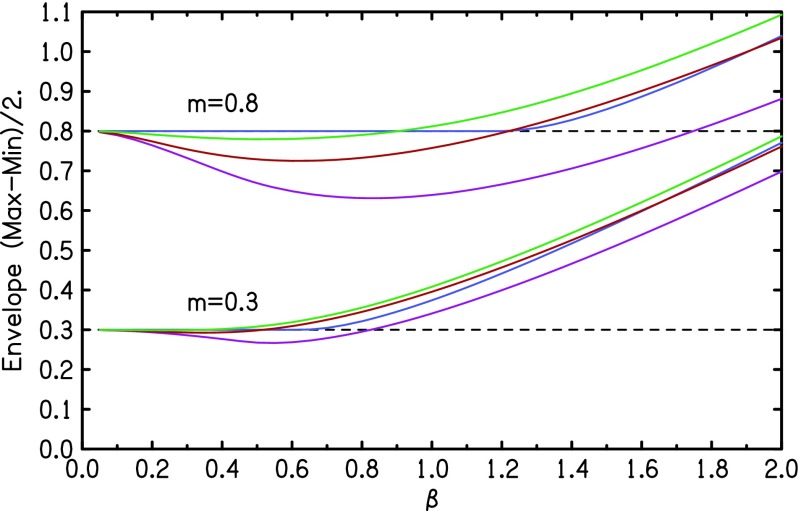
Envelope modulation depth as a function of QFM index *β* for *m* = 0.8 and *m* = 0.3. For each value of *m* there are four plots for $\phi_{f}-\phi_{a}=0$° (blue), 30° (green), 60° (red), and 90° (magenta).

Guidelines indicating the effect of *β* appear in Fig. 4 where the envelope modulation *EM* (expressed as half the difference between the envelope maximum and the envelope minimum) is plotted for two different AM fractions, *m*. The *EM* function is independent of carrier parameters. It depends only on the difference of modulation phases, $\Delta \phi =\phi_{f}-\phi_{a}$ and not on $\phi_{f}$ and $\phi_{a}$ individually. For all *β*, the *EM* function is symmetrical about Δϕ=90°, e.g., it is the same function for Δϕ=60° and Δϕ=120°. For small *β* the difference between *EM* and *m* is greatest for Δϕ=90°. This difference increases as the square of *β* for very small *β* and approximately as the square for moderately small *β*.

The behavior of the *EM* function for Δϕ=0 is very peculiar. If *β* is not large, *EM* appears to be exactly equal to *m*. At some threshold value of *β*, *EM* begins to depart from *m*. The plot for *m* = 0.8 in Fig. 4 is an example. For *β* ≤ 1.20 *EM* equals 0.800000 to six significant figures. But when *β* increases further *EM* departs from 0.8, apparently quadratically in the difference (*β* – 1.20). The threshold decreases for decreasing *m*. For instance for *m* = 0.3 it is *β* = 0.63, as shown in Fig. 4.

## III. EXPERIMENT METHODS

Experiments were done to search for the effects of head diffraction in distorting high-frequency AM tones and on the consequences for localization. The approach combined ear canal measurements using probe microphones (Etymotic ER-7C, Elk Grove Village, IL) with listener localization responses. The experiments presented modulated and unmodulated tones to listeners through 13 loudspeakers, equally spaced over a 90° arc in the right front azimuthal quadrant, in an anechoic room. The loudspeakers were numbered 0 through 12. The experimental setup was the same as that for experiment 1 in Macaulay *et al.* (2010) except for two changes: First, the radius of the loudspeaker array centered on the listener was increased from 112 to 197 cm. Second, a masking noise was presented from a two-way loudspeaker directly behind and beneath the listener in order to mask difference tones (100 and 200 Hz) between the spectral components of the AM signal. The masker noise was played continuously throughout the course of a run. Its spectrum extended from 50 to 250 Hz, and its level was 50 dBC at the listener's head. It was constructed from equal-amplitude, random-phase spectral components having frequencies that were all multiples of exactly 2 Hz. Therefore, in principle, the masking noise could be completely eliminated by matched filtering [e.g., Eqs. (4) and (5)] of a half-second sampled signal. In practice, the matched filtering reduced the residual noise to a negligible size, and the noise did not interfere with the measurements of *A_c_*, *B_c_*, etc.

### A. Stimuli and procedure

There were six experimental stimuli: three unmodulated sine tones (2, 3, and 4 kHz) and three sinusoidally amplitude-modulated tones (SAM tones) with the same carrier frequencies and a modulation rate of 100 Hz (100% modulation). The modulation frequency of 100 Hz is near the region around 128 Hz for which listeners are the most sensitive (Bernstein and Trahiotis, 2002, 2009; Dietz *et al.*, 2013). The 100-Hz modulation frequency was small enough to ensure that all of the spectral components were in the same auditory filter channel for each of the three carrier frequencies as determined by Glasberg and Moore (1990). This feature was implicitly assumed in the description of the spectrum and decomposition in Sec. II, and its importance was remarked by Henning (1980, 1983).^3^ There were 250-ms linear ramps at the beginnings and ends of the signals. The target stimuli had an average level of 65 dBA as measured at the location of the listener. The level on each trial was roved randomly by +2, +1, 0, −1, or −2 dB—enough variation to significantly randomize nonlinear loudspeaker distortion products. Randomization prevented the listener from using level differences or idiosyncratic distortion characteristics to identify sources.

At the beginning of each run, a calibration sine tone from loudspeaker zero (directly in front of the listener) was played while the experimenter viewed the probe microphone signals on an oscilloscope. The experimenter instructed the listener to adjust his or her head to ensure that the fine-structure IPD was as close to zero as possible. This was done under the constraint that the listener felt confident that he or she was facing loudspeaker zero.

A run consisted of five random passes through the 13-loudspeaker array (65 trials). On each trial, there were two identical 1-s tone intervals separated by 1 s, presented by the same loudspeaker. After the second tone, the listener responded verbally with a loudspeaker number. The listener was asked to respond with negative numbers if the source was perceived to be on the left, and with source numbers greater than 12 (or less than −12) if the source was perceived to be behind. Responses “behind” were reflected across the median frontal plane in the final analysis. Each listener completed two runs for each stimulus, which resulted in 10 responses and 20 binaural recordings for each stimulus/loudspeaker combination.

### B. Analysis of signals

The analysis of the recordings was limited to the half second from 256 to 756 ms. This choice eliminated the 250-ms rise/fall times at the beginnings and ends of the signals, and it accounted for the 6-ms delay for the sound to travel from the loudspeakers to the listener. At a sample rate of 50 kHz, each recording contained 25 000 samples per channel. The raw recordings, *x*_raw_, contained electrical noise from the pre-amplifiers, acoustical noise—including the continuous noise of the masker—and distortion. The noise and distortion were almost entirely eliminated by matched filtering of the 0.5-s recording. Using the discrete-time equivalents of the integrals, such as Eqs. (4) and (5), the six A and B coefficients were obtained for each ear for each raw recording, *x*_raw_. These coefficients were then used to calculate the model waveform, *x*_model_, using Eq. (3). The residual noise and distortion was calculated by adding up the squared differences between the raw recording and the model waveform. If the residual noise and distortion exceeded 10%, the recordings and associated listener responses were discarded from further analysis. Out of 3900 trials, only 9 were discarded, usually because the subject was inadvertently talking during presentation. ILDs were calculated from model waveform energies, and subsequently referenced to the ILD at zero azimuth.

The model envelopes were calculated using the matched-filtering coefficients and Eq. (15). Envelope ITDs were calculated using a cross correlation, *γ*(*τ*), as a function of lag time, *τ*, between the left and right model envelopes, *E*_ℓ_ and *E_r_*, respectively,

$$\gamma [\tau ]=\{\begin{array}{cc} \frac{\sum_{t=1}^{25000-\tau }E_{\ell }[t+\tau ]E_{r}[t]}{\sqrt{\sum_{t=1}^{25000}E_{\ell }^{2}[t]\sum_{t=1}^{25000}E_{r}^{2}[t]}}, & \tau \geq 0\\ \frac{\sum_{t=1}^{25000+\tau }E_{\ell }[t]E_{r}[t+\tau ]}{\sqrt{\sum_{t=1}^{25000}E_{\ell }^{2}[t]\sum_{t=1}^{25000}E_{r}^{2}[t]}}, & \tau <0. \end{array}$$ (16)

The value of *γ*[*τ*] at the peak is the envelope coherence, and the corresponding indexed time, *τ*, is the envelope ITD.

### C. Listeners

There were 5 listeners. Listener B was a male aged 59 years. Listeners C, M, and L were males aged 20–25 years. Listener V was a female aged 19 years. All listeners signed a current consent form approved by the Institutional Review Board at Michigan State University. Listeners M, L, and V had normal hearing thresholds within 15 dB of audiometric zero out to 8 kHz. Listener B had a mild hearing loss typical of males his age, but normal thresholds at the frequencies of these experiments. Listener C had normal hearing thresholds except for about 20 dB of hearing loss in his left ear between 1.5 and 4 kHz.

## IV. MEASUREMENTS IN EAR CANALS

The ILD and the envelope ITD are the two cues available to the listener for localization of high-frequency modulated tones.

### A. ILD

**FIG. 5.**
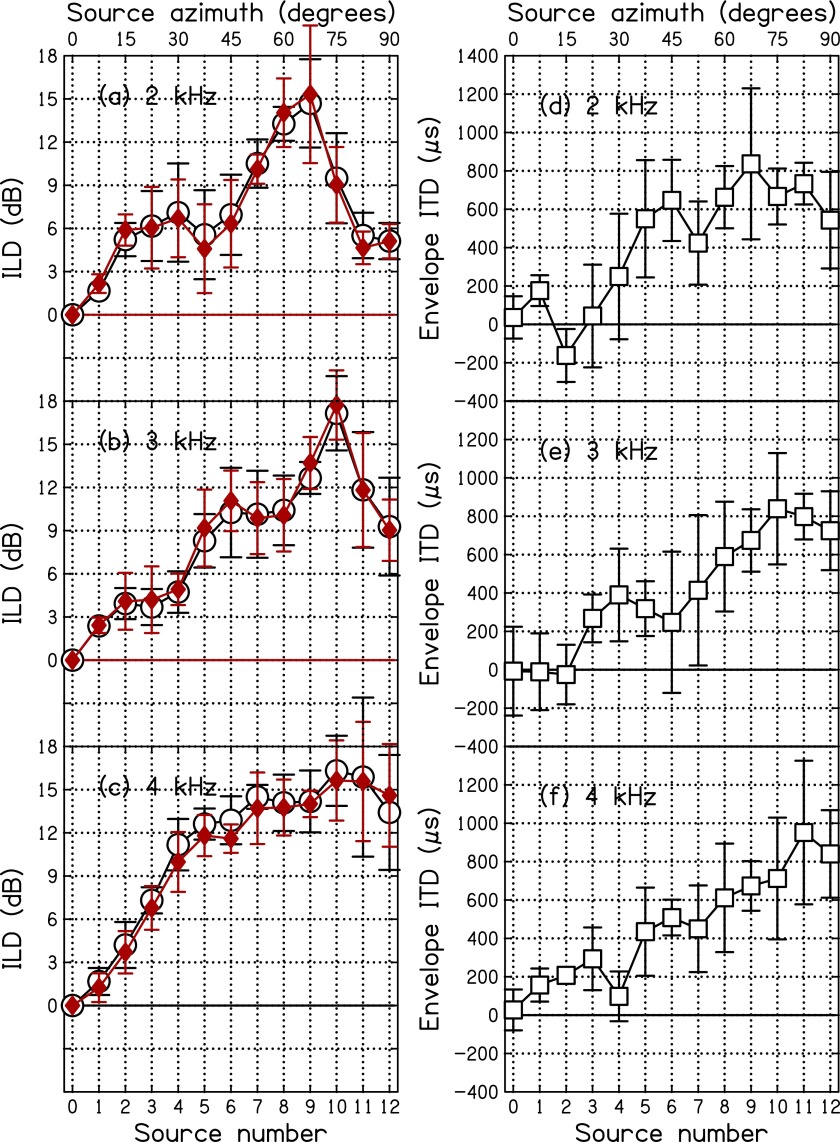
The ILD and EITD for SAM tones are shown by open symbols as functions of source azimuth averaged over the five listeners for the three different frequencies. The ILDs for sine tones are shown by filled diamonds. The error bars are two standard deviations in overall length.

The circles in Fig. 5 show the average ILD values as measured for amplitude modulated tones, averaged over listener. Corresponding average ILD values for sine tones, shown by filled diamonds in Fig. 5 were similar, and both exhibited the effects of the bright spot. The bright spot causes a peak in the ILD function. The peak occurs at increasing values of the azimuth for increasing frequencies. This frequency dependence of the azimuth for which the peak occurs is a general feature of wave diffraction because it can be seen in the spherical head model as well (e.g., Duda and Martens, 1998) as described in detail in Appendix B. The peaked character of the function causes the ILD to be an ambiguous cue for localization (Macaulay *et al.*, 2010). The effects are seen at large source azimuth.

**FIG. 6.**
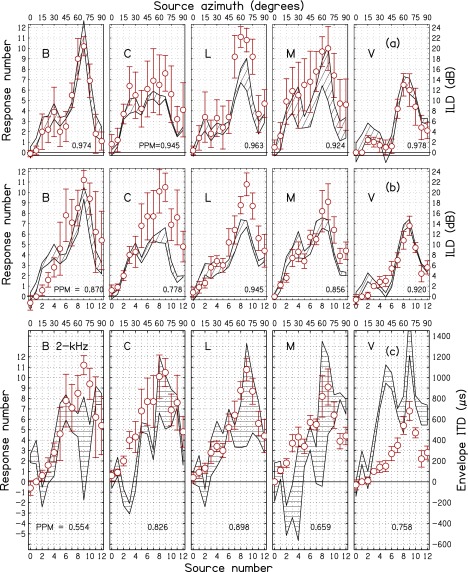
Localization response data (circles) as a function of source azimuth for the five listeners for 2 kHz. The (a) row is for sine tones. The (b) and (c) rows are for SAM tones, and the circles are the same in those two rows. Hatched regions in rows (a) and (b) show ILD. Hatched regions in row (c) show EITD. The error bars on the circles and the widths of the hatched regions are two standard deviations in overall length. PPM in rows (a) and (b) indicate the correlation between responses and ILDs. Correlations are lower for SAM tones as expected. PPM values in row (c) indicate the correlation between responses and EITDs.

**FIG. 7.**
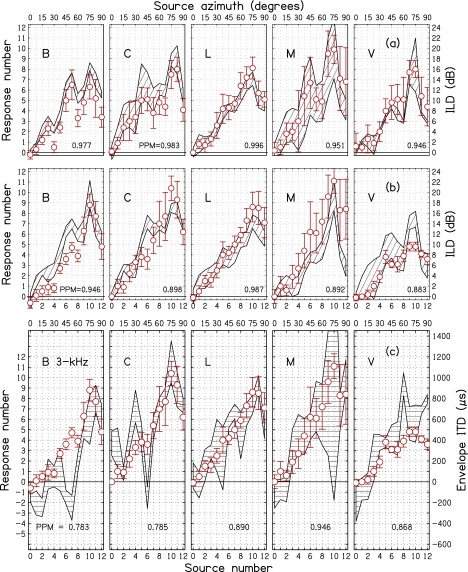
Same as Fig. 6 except for 3 kHz.

**FIG. 8.**
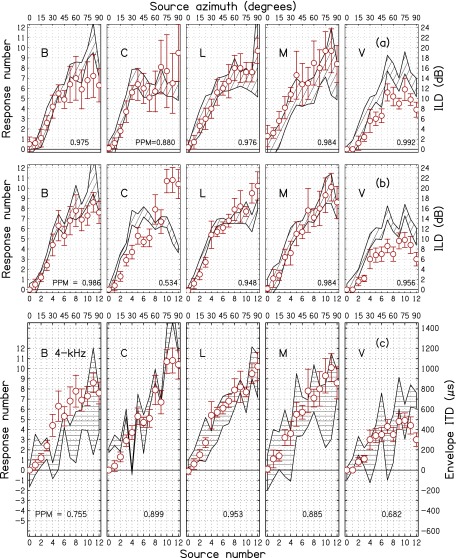
Same as Fig. 6 except for 4 kHz.

ILDs for individuals listeners and frequencies, averaged over trials, are shown by the hatched regions in Figs. 6, 7, and 8 for 2, 3, and 4 kHz carrier frequencies, respectively. The (a) panels show the baseline condition—sine tones and ILD. The (b) panels are for SAM tones and ILD. Because average ILDs were similar for sine tones and SAM tones in Fig. 5, one might expect the ILDs to be the same in (a) and (b) panels, but the addition of sidebands in the SAM tones can have different effects on the ILDs for different listeners, and individual differences appear in the hatched regions, especially for 3 kHz. The visual impression of the differences owes more to the different standard deviations—leading to different widths—than to differences in mean ILDs. Correlations (Pearson product-moments) between the mean ILDs for sine tones (a) and SAM tones (b), averaged across all three frequencies, for listeners B, C, L, M, and V were, respectively, 0.98, 0.95, 0.96, 0.97, and 0.98.

### B. EITD

The EITD was taken to be equal to the lag that maximized the cross-correlation functions in Eq. (16). Envelope ITDs, averaged over listeners, are plotted as functions of source azimuth by squares in Fig. 5. The figure shows that the average EITDs were ragged and not monotonic functions of azimuth. Plots for individual listeners were often even more ragged, as can be seen in Figs. 6, 7, and 8. Figure 5 shows that, similar to the ILD, the EITD also has a maximum as a function of azimuth: at 67.5°, 75°, and 82.5° for 2, 3, and 4 kHz, respectively. Unlike the maximum in the ILD, the maximum in the EITD is not predicted by the spherical head model. Calculations with that model for a source distance of 200 cm show that the EITD continues to grow as the azimuth approaches 90°. However, the nonmonotonic behavior of the EITD for large azimuths is relatively modest and the EITD can be expected to resolve the ambiguity seen in the ILD at large azimuth.

### C. Negative EITD

The squares in Fig. 5 show that some EITDs were negative, even in free field, and even as averaged over listeners. In such cases, the sign of the EITD was opposite to the sign of the phase shift, thus cuing the wrong side. A count—across all listeners, carrier frequencies, and azimuths—found that negative EITDs occurred on 14% of the trials. These negative EITDs were the result of diffraction by the listener's anatomy.

As an extension of our free-field investigation, EITDs were measured in two room environments using a KEMAR manikin (G.R.A.S. Sound and Vibration, Holte, Denmark) and a 4-kHz carrier. The rooms were the lab (Room 10B) and reverberation room described in Hartmann *et al.* (2005). In the lab and the reverb room, the EITD had the wrong sign 22% and 42% of the time, respectively. We conclude that a negative group delay, responsible for the anomalous EITD, was relatively rare (14%) when diffraction alone was involved but became more common when reflections from room surfaces became a major contribution to the sound level.

### D. Envelope shape

By definition, the EITD requires a comparison of equivalent features in the envelopes in the left and right ears. A disparity in the shapes of the envelopes between the two ears represents a potential problem. We chose to quantify such disparity in terms of the interaural envelope coherence—the maximum value of the cross-correlation function from Eq. (16) for all lags (±*τ*), |τ|≤ half a period of the modulation (Aaronson and Hartmann, 2010). To the extent that the auditory system works as a cross correlator of envelopes, this measure of similarity is appropriate.

The interaural envelope coherence measured in free field was found to be surprisingly large. The mean coherence for a given loudspeaker, listener, and carrier frequency, was never lower than 0.94 and most of the measured coherences were greater than 0.99. The mean of the means was 0.996. The coherence tended to be the smallest at azimuths corresponding to small levels in the far ear. The interaural envelope coherence in the reverberation room was smaller than in free field but still large, with a mean of 0.96. Similarly in the lab, the mean was 0.97.

We wondered whether such large values of interaural envelope coherence were peculiar to our conditions or whether they should be expected. Therefore, we performed a computer simulation of *n* = 1000 random pairs of envelopes computed from Eq. (15) with normally distributed *A* and *B* parameters. The simulation found that the interaural envelope coherence did not deviate from unity by much. The mean value was *μ* = 0.96, and the standard deviation was *σ* = 0.03. We concluded that even for random conditions, where envelope shapes may be highly diverse, envelope coherences nonetheless tend to be high.

### E. Modulation percentage

If the modulation percentage in one or both ears is small, the envelope becomes flat, and the concept of EITD loses its meaning. In our free-field conditions—across all listeners, carrier frequencies, and azimuths—the amplitude modulation, *m*, typically varied between 0.7 and 1.5. Compared to the near ear, there was a wider distribution in the far ear, which contained outliers as large as *m* = 3. We wondered about the origin of the observed variation in values of *m*. Some of the deviation from a perfect *m* = 1 originated in the loudspeakers themselves. This deviation was measured for each loudspeaker—alone in an empty anechoic room—using a single microphone (Behringer ECM-8000, Willich, Germany). For the three frequencies the *m* values, averaged across the loudspeakers, ranged from 0.97 to 1.05, and the standard deviation was 0.04 or less. Additional deviation arose from scattering by the array, where measurements found that the mean values of *m* ranged from 0.94 to 1.10, and the standard deviation was 0.11 or less. These deviations could be compared with those found in listener ear canals. There, the distribution of *m* values in the near ear resembled that for the array, but the distribution of *m* values in the far ear showed a larger effect. In the far ear, the mean values of *m* ranged from 0.98 to 1.11, and the standard deviation ranged from 0.17 to 0.34. Therefore, the largest effect on *m* values in our experiment arose from head diffraction.

It is certain that moving the experiment into a room environment would lead to greater variation in the values of *m*, including quite small values. Spot checks in the lab (Room 10B) and reverb room using the KEMAR found that 10% of the *m* values were less than 0.5, but in free field none of them were. Values of *m* as small as 0.13 were sometimes seen in the lab.

For additional comparison, amplitude modulation fractions were measured on a KEMAR wearing *headphones*. For all three of our frequencies, measured *m* values were always within 2% of the expected value of 1.0. This comparison made it evident that real-world listening to 100% modulated signals encounters situations that headphone listening does not.

## V. LISTENER RESPONSES

For nearly every listener and carrier frequency, the response accuracy was better for SAM tones than for sine tones. Response accuracy was first quantified as the correlation between the source azimuth and the response azimuth as averaged over all the trials for a given source. Pearson product-moment (PPM) correlations for each listener and frequency are given in Table I. When SAM was introduced, the average correlation across the three carrier frequencies for listener B increased from 0.72 to 0.88. For listener C, the average increased from 0.73 to 0.84. For listener L, the average increased from 0.83 to 0.90. For listener M, the average increased from 0.80 to 0.87. For listener V, the average increased from 0.79 to 0.83. Response accuracy was next quantified by the rms (root-mean-square) discrepancy between response and source azimuths—also shown in Table I. The rms discrepancy was always smaller for SAM tones than for sine tones, except for listener V at the highest two frequencies. Both measures of response accuracy show that listeners benefited from the AM. Finally, Table I shows the bias, or the mean signed discrepancy between source and response. Nearly every value is negative indicating responses too close to the midline. A detailed analysis showed that the bias arises from the sources at large azimuths where the small ILDs lead to confusion.

**TABLE I.** Response accuracy measures: (1) Pearson product-moment correlations between response azimuths and source azimuths for sine tones and SAM tones. (2) Rms error in degrees comparing response azimuths and source azimuths for sine tones and SAM tones. (3) Error bias in degrees comparing response azimuths and source azimuths for sine tones and SAM tones.

		PPM		Rms error (deg)		Error bias (deg)	
Listener	*f* (kHz)	sine	SAM	Sine	SAM	Sine	SAM
B	2	0.47	0.81	32.2	18.9	−17.8	−7.7
	3	0.78	0.91	28.1	23.0	−21.3	−19.5
	4	0.91	0.94	17.5	13.2	−10.9	−7.5
C	2	0.46	0.73	26.7	19.5	−9.3	−2.4
	3	0.82	0.92	22.8	14.9	−14.7	−9.9
	4	0.91	0.97	14.2	8.0	−6.5	−4.7
L	2	0.63	0.76	24.8	20.6	−7.7	−9.4
	3	0.89	0.97	20.9	15.1	−15.5	−12.3
	4	0.97	0.97	10.3	7.9	−5.0	−3.3
M	2	0.56	0.69	23.6	23.4	−1.2	−11.8
	3	0.88	0.94	15.9	11.1	−8.7	−6.1
	4	0.94	0.97	10.9	8.4	+ 1.0	−3.8
V	2	0.64	0.74	35.3	33.1	−27.9	−26.7
	3	0.85	0.90	22.7	30.7	−16.3	−25.3
	4	0.88	0.84	27.0	30.3	−21.4	−23.8

### A. Responses and interaural cues

The responses, averaged over trials, for individual listeners are shown by circles in Figs. 6, 7, and 8 for 2, 3 and 4 kHz carrier frequencies, respectively. The ILD and EITD are shown by hatched regions with widths of two standard deviations. The (a) panels show the baseline condition—sine tones and ILD. The (b) panels are for SAM tones and ILD. The (c) panels are for SAM tones and EITD. Therefore, the circles in the (b) and (c) panels are the same. Comparison with the circles in the (a) panels shows the effect on responses of adding amplitude modulation.

The tendency for ILD or EITD to drive the localization can be seen by noting how the responses track the hatched regions in Figs. 6, 7, and 8. For instance, at 2 kHz (Fig. 6) the ILD values in panels (a) and (b) for listener C are relatively small. Correspondingly the responses shown in the (a) panel (sine tones) are small. However, upon the introduction of AM, the responses increase substantially, as shown by the circles in the (b) and (c) panels. That indicates that listener C was affected by the EITD. Listener V also experienced small ILDs and produced small responses for sine tones as shown by the circles in panel (a). However, panels (b) and (c) show that responses did not increase upon the introduction of AM. That indicates that listener V was not much affected by the EITD.

Figures 6, 7, and 8 suggest that responses follow the ILD better than they follow the EITD. This becomes particularly apparent on comparing the PPM values in panels (a) (for ILD) and (c) (for EITD) of these figures, where 14 out of the 15 correlations were higher for ILD than for EITD. Nevertheless, one would expect that the EITD is responsible for changing listener responses when amplitude modulation is introduced because the ILD has changed very little and randomly, whereas the EITD is a new, systematic cue. One simple test of the effect of EITD is to examine the effect of negative EITDs. Focusing on the 14% of EITDs that were negative, we found that 79% of these lead to decreased laterality compared to sine tones with no modulation.

**FIG. 9.**
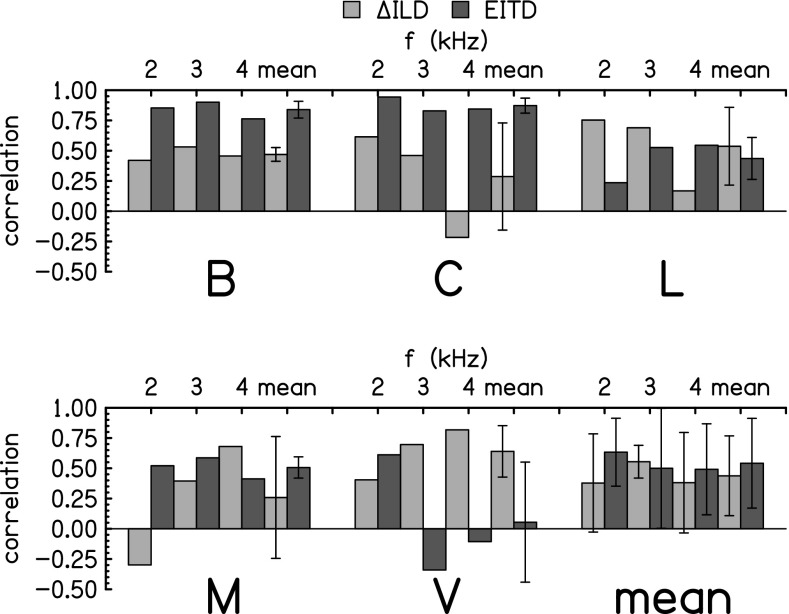
PPM correlation coefficients for the change in response attributable to AM and the change in ILD are shown by light grey bars. Correlation coefficients for the change in responses and the EITD are shown in by dark grey bars. For each listener, the three frequencies and the mean and standard deviation across frequencies are shown. The plot in the lower right averages across all the listeners. The error bars are two standard deviations in overall length.

To further test the importance of the EITD, we investigated the correlations between the *change* in listener response (AM–sine) and the changes in stimulus cues. Changes in stimulus cues were (fortuitous) changes in ILD and the introduction of the EITD itself. The results are shown in Fig. 9 for all listeners and frequencies. Extensive calculations also computed the correlations between the change in listener response and the change in *compressed* cues (Macaulay, 2015).

The correlations in Fig. 9 suggest that listeners B and C (and possibly M) were strongly influenced by the introduction of the EITD cue, but that the other listeners were not. The compressed cue calculations lead to the same conclusions. Because of this difference in correlations, one might expect that listeners B and C would have benefited the most when sine tones were replaced by SAM tones. One might also expect that listener V would have benefited the least. These expectations agree with the improvements in response accuracy, as reported in Table I. For instance, the correlations between responses and azimuths averaged over frequency (see the first paragraph of Sec. V) increased by 0.16 and 0.11 for listeners B and C; 0.07 for listeners M and L, but only 0.04 for listener V. Similarly, the rms discrepancy averaged across frequency was decreased by SAM tones: 7° for listeners B and C; 4° for listener L; 2° for listener M, but −3° for listener V.

### B. Response changes and mixed modulation parameters

The changes in response accuracy and the correlations in Fig. 9 show that some listeners benefited from the introduction of a modulated envelope ITD much more than others. One possible reason for this difference is that the listeners who benefited more received modulation cues that were more useful, presumably because of anatomical differences. Another possibility is that the modulation cues were physically of similar quality for all listeners, but some listeners were more sensitive to these cues and better able to take advantage of them. This section tries to decide between these alternatives by comparing individual changes in responses caused by AM with individual modulation parameters. Four parameters were identified as likely sources of individual differences: EITD quality, modulation percentage in the far ear, modulation percentage in the near ear, and interaural envelope coherence. We conjectured that the differing quality of these parameters might have caused the response differences.

#### 1. EITD Quality

As shown in Figs. 5(d), 5(e), and 5(f) and in Figs. 6(c), 7(c), and 8(c), the EITD is a ragged function of azimuth. We conjectured that the EITD might be a more reliable guide to azimuth for listeners B and C who benefited most from AM and a worse guide for listeners L, M, and V who benefited least. However, we found no support for that conjecture in the measured correlations between EITD and source azimuth. The correlations for listeners B and C were actually below the average over listeners and the correlations for listeners L and M were above average.

As noted in the Introduction, negative EITDs are a source of possible confusion. In fact, when negative EITDs occurred, they caused the response azimuth to decrease the great majority of the time, as would be expected. However, negative EITDs did not occur more frequently for the listeners who benefited least. For listeners B, C, L, M, and V, the numbers of negative EITDs was 8, 5, 2, 8, and 5, respectively.

#### 2. AM Quality

The assessment of AM quality began with plots of the *change* in response caused by the introduction of amplitude modulation as a function of the measured EITD. With five listeners and three frequencies, there were 15 such plots. Slopes and intercepts given by linear regression for all 15 plots are given in columns (a) and (b) of Table II. Averaged across the three frequencies, the slopes show the expected large differences between listeners: 24° and 29°/ms for B and C (who benefited most), 6° and 7°/ms for L and M, and finally 2°/ms for V (who benefited least).

**TABLE II.** This table refers to plots of the *change* in listener response, caused by the introduction of AM, vs the EITD (plots not shown). Columns a and b are parameters for the best fit straight line to these plots. Columns c, d, and e show correlations between the residuals, namely, the difference between responses and the best fit line, and three different AM parameters. Correlations are PPM and are reported for each listener and frequency.

		a	b	c	d	e
Listener	*f* (kHz)	Slope (°/ms)	*y* intercept (°)	*m* left	*m* right	Envelope coherence
B	2	42.7	−7.9	−0.06	−0.23	−0.06
	3	23.6	−1.6	0.04	0.11	−0.33
	4	4.4	2.0	0.25	0.13	−0.23
C	2	34.9	−5.0	−0.12	0.10	−0.18
	3	27.0	−9.8	−0.03	0.19	0.24
	4	24.6	−14.4	0.02	−0.46	0.20
L	2	4.0	−3.3	−0.35	0.15	−0.26
	3	6.1	0.6	0.23	−0.01	−0.18
	4	8.5	−2.7	0.27	−0.01	−0.13
M	2	7.5	−12.9	0.01	0.01	0.04
	3	7.7	−5.0	0.03	0.16	−0.01
	4	5.8	−7.4	0.10	−0.10	−0.13
V	2	9.5	−4.7	−0.13	−0.05	0.17
	3	−5.5	−6.8	−0.23	0.01	0.02
	4	1.1	−2.8	−0.42	−0.21	0.27

We considered an AM-quality hypothesis predicting that trials with lower quality AM will tend to fall below the linear regression line because the EITD will be less effective in causing a change if the AM quality is low. Similarly, trials with higher quality AM will tend to fall above the line of best fit. This hypothesis arose from the fact that average responses for sine tones underestimated the source azimuth.

A quantitative evaluation of the AM-quality hypothesis is the correlation between the residuals and the different modulation parameters for those plots. Columns (c), (d), and (e) of Table II give a summary of the correlations for each listener and frequency for *m*-far, *m*-near, and envelope coherence. One would expect these correlations to be positive, because a more effective EITD should increase the average response azimuths. However, there are about as many negative correlations as there are positive, and none of them are large.

Table II shows no correlation between response changes and the preservation of modulation fractions, *m*. A possible explanation for this negative result is that the values of *m*, as they occurred in free field, were never really low enough to compromise the use of the EITD. Nuetzel and Hafter (1981) showed that for 4-kHz SAM tones modulated at 150 and 300 Hz, the just-noticeable-difference (JND) in the EITD does not vary greatly with *m* when *m* is greater than about 0.5—a range that includes all our measured values in free field.

#### 3. Responses for large azimuths

It is possible that the lack of significant correlations found in Sec. V B 2 was caused by the large number of sources at small azimuths for which the ILD provided a reliable cue, and for which the EITD did not contribute new and different information. Including these small azimuth sources might be expected to reduce correlations because the responses did not change much for them. Therefore, we considered AM-induced changes in listener responses for large azimuths.

For azimuths greater than 60° (loudspeakers 8 through 12) the bright spot led to anomalous ILD.^4^ For these azimuths, EITDs were large and they usually contradicted the trend of the ILDs. Here, the listener response azimuths for sine tones were always smaller than the true azimuths, and a positive effect of the EITD would then make the response azimuths larger and in better agreement with reality.

Looking only at responses changes for these large azimuths, we computed correlations with modulation fractions in near and far ears, both the values and deviations from 1.0. The absolute value |m−1| was thought to be particularly important because *m* values larger than 1 and smaller than 1 both reduce the rising slope of the ongoing modulated envelope and reduce the “off-time” or “pause”—known to be important features perceptually (Dietz *et al.*, 2015). We also computed the correlation with envelope coherence. None of these correlations proved to be significant, and most of them were both small and negative.

## VI. DISCUSSION

Compared to the large number of headphone studies of the effects of amplitude modulation on lateralization, there are rather few free-field studies. The letter by Eberle *et al.* (2000) describes free-field experiments using an octave band of noise, 7–14 kHz. Eberle *et al.* found no advantage to sound localization when amplitude modulation was introduced. However, the virtually-localized headphone experiments with broadband noise by Macpherson and Middlebrooks (2002) found that modulation led to an increase in weight given by listeners to the ITD cue. Our experiments found that introducing amplitude modulation almost always improved the localization of high-frequency tones with slow onsets. As suggested by Eberle *et al.*, it is likely that the fluctuations in the noise in their experiments provided a useable envelope timing cue so that the introduction of modulation provided no measurable additional benefit. By contrast, our unmodulated tones provided no useable timing cues at all, and the introduction of modulation provided a qualitatively new localization cue.

While this article has mainly concerned the physical effects of head diffraction on the availability of envelope timing cues for sound localization, it is worth noting that there are other effects that potentially compromise the effectiveness of amplitude modulation. As noted by Dietz *et al.* (2013) passing a SAM signal through an auditory filter having a bandwidth less than the modulated signal bandwidth leads to a reduced modulation fraction. For a modulation frequency of 100 Hz, the bandwidth is 200 Hz, and for a carrier frequency of 2 kHz, the equivalent rectangular bandwidth obtained by Glasberg and Moore (1990) is 245 Hz. Applying a fourth-order gammatone filter with this bandwidth reduces a modulation fraction of 1.0 to only 0.73—a major effect. By contrast, for a carrier at 4 kHz, the fraction is only reduced to 0.92. To the extent that this effect on modulation fraction is major, one would expect a greater sensitivity to EITD for a 4-kHz carrier than for a 2-kHz carrier. However, our experiments found that the correlation between listener response change and EITD showed no significant frequency effect. Also, the headphone experiments by Dietz *et al.* (2013) (4-kHz carrier and modulation frequencies of 32 and 128 Hz) showed an opposite effect. They found that ITD thresholds were always higher for 32 Hz than for 128 Hz—by as much as a factor of 4. In the end, we have no evidence for an effect of auditory filtering per se on the benefit of EITD.

Yost and Zhong (2014) studied the effect of bandwidth on the ability of listeners to localize noise bursts in the azimuthal plane. Center frequencies of 2 and 4 kHz were included, similar to our work. Although there was no explicit amplitude modulation, the different bandwidths led to amplitude fluctuations that can be related to modulation frequencies using a formula from Rice (1945), namely, that the expected number of envelope maxima per second is 0.6411 times the bandwidth of an ideal filter. Therefore, the effective modulation frequencies at 2 kHz ranged from 44 to 297 Hz, and those at 4 kHz were twice as high. Although the expected fluctuation rates became much larger than auditory filter widths, localization error rates decreased monotonically with increased bandwidth as measured in octaves. Within the error bars, combining results for the two frequencies, error rates also decreased with increasing bandwidth as measured in Hertz. Therefore, it appears that wide bands of noise are well localized because a larger bandwidth provides more information about ITD (possibly also ILD) to the auditory system.

Dynamic range compression in the auditory system is a second mechanism whereby the peak to trough difference in a modulated signal is reduced, effectively reducing the modulation fraction. There are critical questions about processes and time constants. The temporal properties of neurons throughout the auditory system exhibit a range of time constants. The longer time constants become evident in modulation transfer functions (Viemeister, 1979) indicating reduced sensitivity as the modulation frequency approaches 100 Hz. Sluggishness like this can be contrasted with the automatic gain control in the cochlear amplifier that is certainly fast enough to attenuate the peaks and increase the valleys of a tone modulated at only 100 Hz (Yates, 1990). This nonlinear mechanism also reduces the effective modulation fraction.

## VII. SUMMARY AND CONCLUSION

For more than 50 years, it has been known that human listeners are able to use the EITD as a cue to the location of sine-wave amplitude modulated (SAM) tones. The EITD provides a useable temporal cue even for tones with frequencies above 1400 Hz, where the human binaural system is unable to process ITD in the fine structure. Over the course of half a century, much has been learned about the processing of EITD at high frequency. For instance, Buell *et al.* (2008) showed that EITD from SAM in the ongoing signal is perceptually more important than interaural time differences in the onset envelope. Headphone experiments by Monaghan *et al.* (2013) showed that simulated reverberation had a greater deleterious effect on high-frequency EITD discrimination than on low-frequency ITD discrimination. The EITD is particularly important in electric hearing because contemporary encoding strategies eliminate the fine-structure ITD from cochlear implants. In that connection, transposed stimuli (Bernstein and Trahiotis, 2002) and exponentiated-offset AM (Bernstein and Trahiotis, 2012) are relevant extensions of SAM. All of this work has been done with headphone listening.

The present article, extended the study of SAM tones to free-field listening. When SAM tones are subjected to diffraction by the human head, the proximal stimulus is changed: AM is turned into mixed modulation, envelopes in the two ears acquire different shapes, and dispersion leads to occasional negative group delays. These effects can be expected to make high-frequency EITD localization difficult. At the same time, localization by means of EITD becomes more important at high frequencies because the only other available cue is the ILD, and the ILD is a confusing, non-monotonic function of azimuth because of the bright spot (Macaulay *et al.*, 2010).

This article began by solving the experimental problem of determining the modulated waveform in the two ear canals. A SAM stimulus leads to six measurable spectral coefficients in each canal which, in turn, determine the six parameters of the mixed modulation. Section II presented a mathematical transformation from coefficients to parameters with six coupled equations. Sections III and IV used ear canal measurements on five listeners to show that all the modulation distortions expected from ordinary head diffraction actually do occur in free field. Further measurements in rooms showed that the difficulties become much worse in reflective environments.

Section V presented the results of localization experiments for pure tones and SAM tones using three carrier frequencies: 2, 3, and 4 kHz. The dominant role of the ILD, including the effect of the bright spot, was apparent in the data with and without AM. Listener responses almost always correlated better with the ILD than with the EITD. The data also showed that, despite the distortions introduced by head diffraction, our listeners localized better with SAM than without it (i.e., than with pure sine tones). Improvements were seen in a reduced influence of the ILD bright spot. However, the improvements in localization were quite different for different listeners. We conjectured that some listeners benefited from SAM more than others because the AM cues were better preserved for these listeners—the AM-quality hypothesis. Given the carrier frequencies and the azimuths involved (0° to 90° in 7.5° increments) some head geometries might be better than others in preserving the relevant modulation cues to EITD.

To test the AM-quality hypothesis, we compared the change in localization attributable to SAM with modulation parameters measured in the ear canals. We considered EITD quality, modulation fractions in near and far ears and interaural envelope coherence. We separately considered parameters for all the sources and for the critical sources at large azimuths. We considered the absolute deviation of the modulation fraction from 1.0 and minima of that fraction. We also considered the quasi FM index (*β*), the overall improvement in response accuracy attributable to AM, and other, more fanciful hypotheses not presented here. However, none of our attempts to associate individual improvement in localization with modulation parameters led to a notable correlation. In the end, this research concludes that individual differences regarding the benefit of added amplitude modulation were not caused by differences in head diffraction causing differences in the proximal stimuli. We interpret the observed individual differences as the result of different processing abilities—some listeners were better able than others to take advantage of the EITD in SAM signals.

This report has been exclusively concerned with the linear distortion of the physical EITD by the listener's anatomy and environment. However, the envelope time difference that is relevant to the listener is a comparison between neural spike trains as they arrive at a central site. The neural spike trains differ from the physical signals at the ear drums by the highly nonlinear processes of compression and spike generation (e.g., Wang *et al.*, 2014). In addition are those operations of the basilar membrane in the cochlea that are normally treated theoretically as linear filters. Linear cochlear filtering produces changes in the envelopes, similar to anatomical diffraction. A common theoretical simplification assumes that left and right cochleae are identical and that interaural differences are compared only within corresponding cochlear channels (Colburn, 1973, 1977). Within that assumption, cochlear filtering can deform envelopes, but left and right channels would be deformed in the same way, unlike the interaural differences that result from anatomical diffraction.

The work described in this report can be regarded as a first step in the study of localization (as opposed to lateralization) of modulated tones. Ear canal measurements showed that our free-field experiments led to relatively modest changes in the EITD. Head diffraction by itself leads to significant degradation of modulation parameters, but not dramatic degradation. By contrast, reflections in ordinary room environments do lead to dramatic changes. The effects were seen in the headphone simulations by Monaghan *et al.* (2013). Standing waves in rooms, coupled with head diffraction, can be expected to make the EITD less useful as a guide to source azimuth. Interestingly, the experimental procedures described in this article, including the mathematical transformation from spectral parameters to mixed modulation parameters, could be taken over entirely without modification to a similar study of EITD localization in reflective environments. In view of the importance of EITD localization and the ubiquity of reflective environments such an extension would be a useful one.

## Figures and Tables

**FIG. 10.**
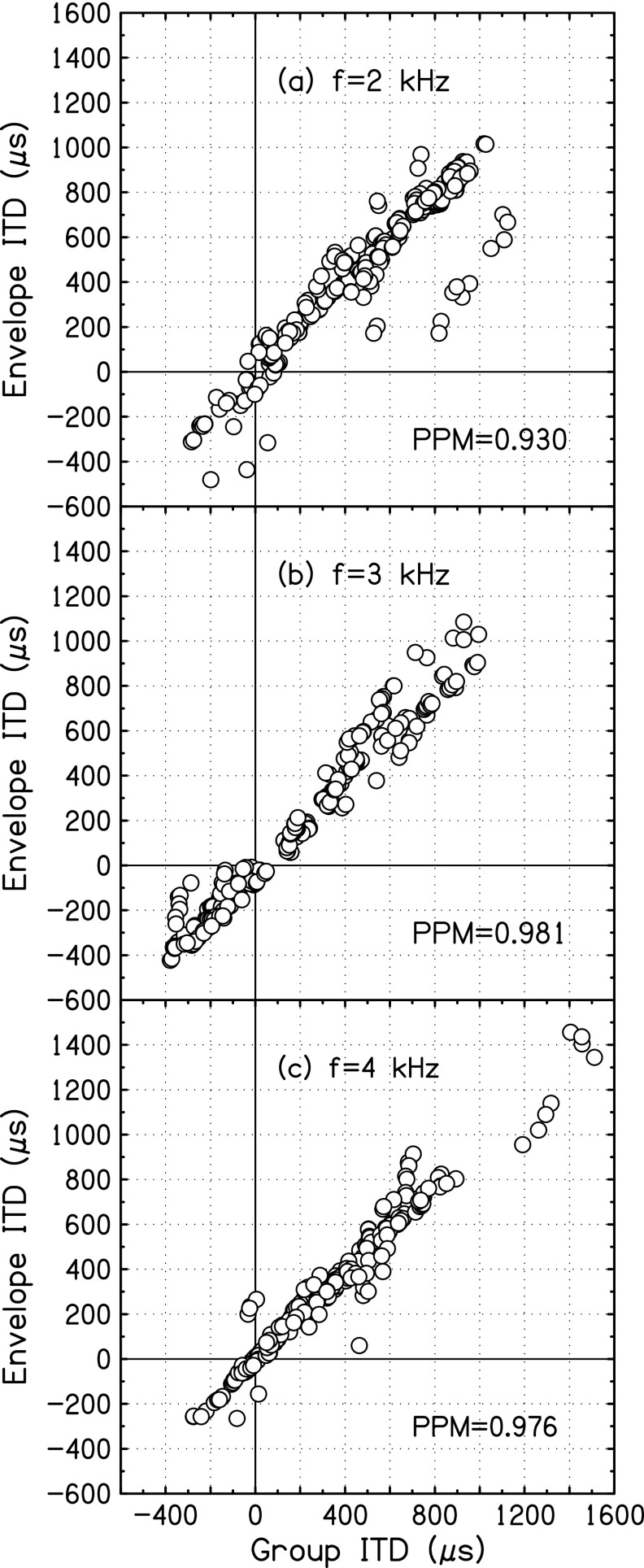
Scatterplot of envelope ITD (EITD) vs group ITD (GITD) for AM tones in the ear canals of listener B. Each panel shows 260 points, 13 sources × 20 tones. PPM correlation coefficients are all greater than 0.9.

## Footnotes

^1^ Although the electrical signal was perfect AM, an imperfect loudspeaker response (amplitude and phase) led to some mixed modulation. See Sec. IV E.

^2^ Beyond the QFM as it appears in Eq. (2), there is one other term that is first order in *β*, namely, the term proportional to *mβ*. That term leads to second sidebands, but these will not occur in a linear system. That term also leads to a small correction in the carrier amplitude, and that is ignored in our treatment.

^3^ Nevertheless, the 200-Hz bandwidth of the AM signal is wide enough to experience auditory filtering, as described in Sec. VI. The effect of auditory filtering could be easily incorporated into the mathematical formulation of this article because both amplitude and phase effects are naturally represented by the Fourier coefficients *A_c_*, *B_c_*, etc.

^4^ Figures 6–8 indicate that the azimuth of the ILD peak increases with increasing frequency in the range of our experiments. Therefore fewer sources lead to ILD confusion for increasing frequency. See Appendix B for a model comparison.

## APPENDIX A: GROUP ITD AND ENVELOPE ITD

This appendix is devoted to the connection between the group interaural time difference (GITD), as it appears in Fig. 2, and the EITD, where the envelope is defined by Eq. (16). The EITD is the standard interaural delay in the binaural literature, and it is the interaural delay used for AM envelope data in this article.

Figure 2 shows the GITD as a derivative of an interaural phase with respect to frequency. However, for a sine-wave modulated tone, the interaural phase is defined only at the three frequencies of the spectral components, and the GITD must be defined through finite differences. To establish a unique GITD, this appendix ignores the phase of the carrier component and defines the GITD in terms of the interaural phase difference for upper and lower sidebands, i.e., $\text{GITD}=(\Delta \phi_{u}-\Delta \phi_{\ell })/(2f_{m})$, where 2 *f_m_* is the frequency separation between the sidebands.^5^

The GITD is similar to the EITD, but the two measures are not generally the same. There are interesting special cases in which these two interaural differences are identical. (1) If one begins with a perfect AM signal in both ears, where the signal is delayed in one ear with respect to the other, the GITD equals the EITD, and both are equal to the signal interaural delay. It is not necessary for the modulation fraction *m* to be the same in both ears. (2) If one then randomizes all the spectral amplitudes but leaves the phases unchanged (remembering that amplitudes are non-negative by definition) then the GITD continues to equal the EITD, which is the signal interaural delay (3). Alternatively, if one begins with a perfect AM signal in both ears, where the signal is delayed in one ear with respect to the other, and randomizes all the phases but leaves the upper and lower sideband amplitudes equal to each other in the left ear and, independently, leaves the upper and lower sideband amplitudes equal to each other in the right ear, then the GITD continues to equal the EITD, but not generally equal to the signal delay because of the change in component phases.

Apart from the special cases in which the GITD and the EITD are identical, there are many cases where they are similar. Therefore, it is not surprising to find that in practical situations, a scatter plot of EITD vs GITD is mainly along the diagonal. Figure 10 shows such a scatter plot for the signals in the ear canals of listener B at the three different frequencies. For 2, 3, and 4 kHz, the best fit lines have slopes of 43°, 43°, and 45°, respectively. Plots for other listeners were similarly concentrated near the diagonal, but they differed in some details. For instance, plots for other listeners did not all show that the largest departures from the diagonal occurred for 2 kHz, nor did they all show that the largest GITD and EITD values occurred for 4 kHz.

## APPENDIX B: SPHERICAL HEADS AND THE ILD

The ILD is a non-monotonic function of source azimuth because of diffraction by the head. The most notable effect is the bright spot at the far ear when the source is directly opposite that ear (i.e., when the source is 180° away from the ear). The bright spot at the far ear causes a minimum in the ILD function. Therefore, the ILD shows a peak as a function of azimuth.

The effects of the bright spot can be calculated analytically for a spherical head, with results that compare reasonably with the more realistic shapes used by Cai *et al.* (2015). Table III shows *θ*_peak_, the azimuth of a source for which the peak occurs, measured with respect to the forward direction of the head. Peak azimuth *θ*_peak_ is calculated as a function of frequency for a source that is far from the head (infinite distance limit). Then the only model parameters are the sphere radius (here 8.75 cm), the speed of sound (34 400 cm/s), and the ear angle. The ear angle, *θ*_ear_, describes the location of the ears on the spherical head, measured as an angle from the forward direction. The left columns of Table III are for an ear angle of 90°; the right columns are for 100°.

**TABLE III.** ILD peak parameters for ear angles of 90° and 100° in the spherical head model. Parameter *θ*_peak_ is the source azimuth for the peak relative to the forward direction. (The ear locations are symmetrical with respect to the forward direction.) “Height” is the difference between the absolute peak height and the minimum at the bright spot.

	*θ*_ear_ = 90°		*θ*_ear_ = 100°	
*f* (kHz)	*θ*_peak_ (°)	Height (dB)	*θ*_peak_ (°)	Height (dB)
1	41	2.6	33	2.0
2	57	6.3	47	5.9
3	66	9.2	56	8.8
4	71	11.0	61	10.9
5	75	12.4	65	12.2
6	77	13.6	67	13.6
7	79	14.5	69	14.4
8	80	15.5	70	15.4

Mathematically, an ILD peak occurs even for low frequencies. For instance, for an ear angle of 90° and a frequency of 450 Hz, an ILD peak occurs at a source azimuth of *θ*_peak_ = 71°. As the frequency increases, *θ*_peak_ decreases rapidly, reaching a minimum of 39° when the frequency is 900 Hz. However, for these low frequencies the peak is small. The peak becomes an important effect only for frequencies near 1 kHz and above. For these higher frequencies, *θ*_peak_ increases monotonically with increasing frequency. Table III shows that the effect of a 10° increase in ear angle is simply a shift of 10° in *θ*_peak_, as expected from simple geometry, except at 1 kHz.

Entries in the “Height” column of Table III show the difference between the peak ILD and the minimum ILD at the bright spot. This minimum occurs at an azimuth of 90° or 80° for ear angles of 90° or 100°, respectively. The height of the peak (in dB) is a quantitative way to estimate the confusion caused by the bright spot.

To summarize, spherical head calculations show two effects as the frequency increases: there is a smaller range of source azimuths leading to confusion, but the amount of confusion caused by any single such source increases. The ILD data in Figs. 6–8 show that the former effect dominated our measurements on human listeners.

Calculations were also done for finite source distances. Interestingly, the height of the peak (as defined here) is always smaller when the source distance is finite, but that effect tends to go away as the frequency increases. Making the source distance as small as 1 m, causes the height of the peak to decrease by 0.5 dB or less when the frequency is 2 kHz or greater. The decrease in height of the peak for smaller source distance occurs because the minimum ILD (at the bright spot) is always larger for smaller source distance. Hence the difference between the absolute height of the peak and the minimum ILD is always smaller.
